# Kansas

**Published:** 1897-08

**Authors:** 


*Kansas.*—The experiment is about to be undertaken of allowing cattle from the nineteen counties in Texas above the quarantine-line to be dipped for the destruction of the ticks, and then allowed the privilege of grazing on Kansas pasture-fields. Whether this will be followed by any outbreaks of Texas fever will be watched with much interest.

Texas fever has broken out on a farm near Manhattan. Some twenty-five animals have died and many others are infected. Rigid control measures and a complete quarantine has been declared.

The Kansas Live-stock Sanitary Commission requires certificate of health, showing freedom from Texas-fever, for cattle shipped from a large number of counties of Texas and Oklahoma.

